# DDA suppresses angiogenesis and tumor growth of colorectal cancer *in vivo* through decreasing VEGFR2 signaling

**DOI:** 10.18632/oncotarget.11152

**Published:** 2016-08-09

**Authors:** Shiu-Wen Huang, Jin-Cherng Lien, Sheng-Chu Kuo, Tur-Fu Huang

**Affiliations:** ^1^ Graduate Institute of Pharmacology, College of Medicine, National Taiwan University, Taipei, Taiwan; ^2^ Graduate Institute of Pharmaceutical Chemistry, China Medical University, Taichung, Taiwan

**Keywords:** angiogenesis, anthraquinone, endothelial cells, VEGFR

## Abstract

As angiogenesis is required for tumor growth and metastasis, suppressing angiogenesis is a promising strategy in limiting tumor progression. Vascular endothelial growth factor (VEGF)-A, a critical pro-angiogenic factor, has thus become an attractive target for therapeutic interventions in cancer. In this study, we explored the underlying mechanisms of a novel anthraquinone derivative DDA in suppressing angiogenesis. DDA inhibited VEGF-A-induced proliferation, migration and tube formation of human umbilical vein endothelial cells (HUVECs). DDA also reduced VEGF-A-induced microvessel sprouting from aortic rings *ex vivo* and suppressed neovascularization *in vivo*. VEGF-A-induced VEGFR1, VEGFR2, FAK, Akt, ERK1/2 or STAT3 phosphorylation was reduced in the presence of DDA. In addition, NRP-1 siRNA reduced VEGF-A's enhancing effects in VEGFR2, FAK and Akt phosphorylation and cell proliferation in HUVECs. DDA disrupted VEGF-A-induced complex formation between NRP-1 and VEGFR2. Furthermore, systemic administration of DDA was shown to suppress tumor angiogenesis and growth in *in vivo* mouse xenograft models. Taken together, we demonstrated in this study that DDA exhibits anti-angiogenic properties through suppressing VEGF-A signaling. These observations also suggest that DDA might be a potential drug candidate for developing anti-angiogenic agent in the field of cancer and angiogenesis-related diseases.

## INTRODUCTION

Angiogenesis, a new blood vessel forming process, consists of basement membrane degradation, endothelial cell migration, proliferation and formation of tubular structures. It participates in various pathological events such as diabetic retinopathy, arthritis and malignancy beyond its role in physiological processes [1, 2]. Given the key role of angiogenesis in tumor progression [3], targeting angiogenesis to destruct tumor vasculature thus represents a promising strategy to suppress tumor progression [3, 4]. Tumor cells or tumor micro-environment promotes angiogenesis through the induction of various cytokines or growth factors such as vascular endothelial growth factor (VEGF), angiopoietin, basic fibroblast growth factor (bFGF) and epidermal growth factor (EGF)[5]. The VEGF family member VEGF-A contributes mainly to tumor angiogenesis and elevated VEGF-A level is associated with tumor progression [6, 7].

VEGF-A augments serial steps of angiogenesis such as increasing vascular endothelial cell proliferation, migration and vascular permeability [8]. VEGF-A exhibits these angiogenic properties through the binding and activation of receptor tyrosine kinases known as VEGFR-2/Flk-1/KDR [9]. VEGF-A binds to VEGFR2 and thereby activates several signaling pathways such as Src, extracellular signal-regulated kinases (ERK), phosphoinositide 3-kinase (PI3K), focal adhesion kinase (FAK) and protein kinase B (Akt) [10]. These signaling cascades stimulate endothelial cell survival, migration, proliferation and forming tubular structures [11–13]. In addition, VEGF-A signaling pathway also involves signal transducers and activators of transcription proteins (STATs) [14]. Yahata Y. et al. [15] demonstrated that STAT3 is required for VEGF-A-induced angiogenesis. VEGF-A-VEGFR2 signaling thus represents a promising therapeutic target in suppressing angiogenesis or angiogenesis-related diseases [16].

Currently, the assessment of various strategies that interfere with VEGF-A-VEGFR2 in clinical trials are undertaken. For instance, small molecule inhibitors that suppress VEGFR2 kinase activity [17], soluble receptors that sequester VEGF-A [18] and antibodies targeting VEGF-A or VEGFR [19]. To date, the United States Food and Drug Administration (FDA) has approved the clinical use of monoclonal antibody such as bevacizumab (Avastinâ) [20] and small molecule inhibitors such as sorafenib (Nexavarâ, BAY 43-9006) in the treatment of treating certain types of cancers [20]. However, adverse effects with bleeding-related complications of most receptor tyrosine kinase inhibitors have been reported [21]. Therefore, developing novel anti-angiogenesis agents with milder side effects is still being investigated [22].

Anthraquinone derivatives exhibit broad pharmacological properties and have been extensively investigated. Recent studies reported their potential use as anti-tumor agents [23–25]. They also exhibit anti-angiogenic properties [26], but the mechanisms for this effect remain incompletely understood at this time. Given their potential as lead compound for drug discovery, we explored the anti-angiogenic mechanisms of a novel anthraquinone derivative DDA (1,5-dihydroxy-4,8-dinitro anthraquinone). We demonstrated that DDA inhibited VEGFR2 signaling and subsequent angiogenesis in VEGF-A-stimulated HUVECs. In Matrigel plug models, DDA suppressed VEGF-A- and breast cancer cell line MDA-MB-231cells-induced angiogenesis *in vivo*. Furthermore, DDA also reduced HCT116 xenograft growth. Taken together, we demonstrated in this study that DDA might be a potential therapeutic agent in suppressing angiogenesis and consequent tumor progression.

## RESULTS

### DDA suppressed HUVEC proliferation, migration and invasion in response to VEGF-A

**Figure 1:**
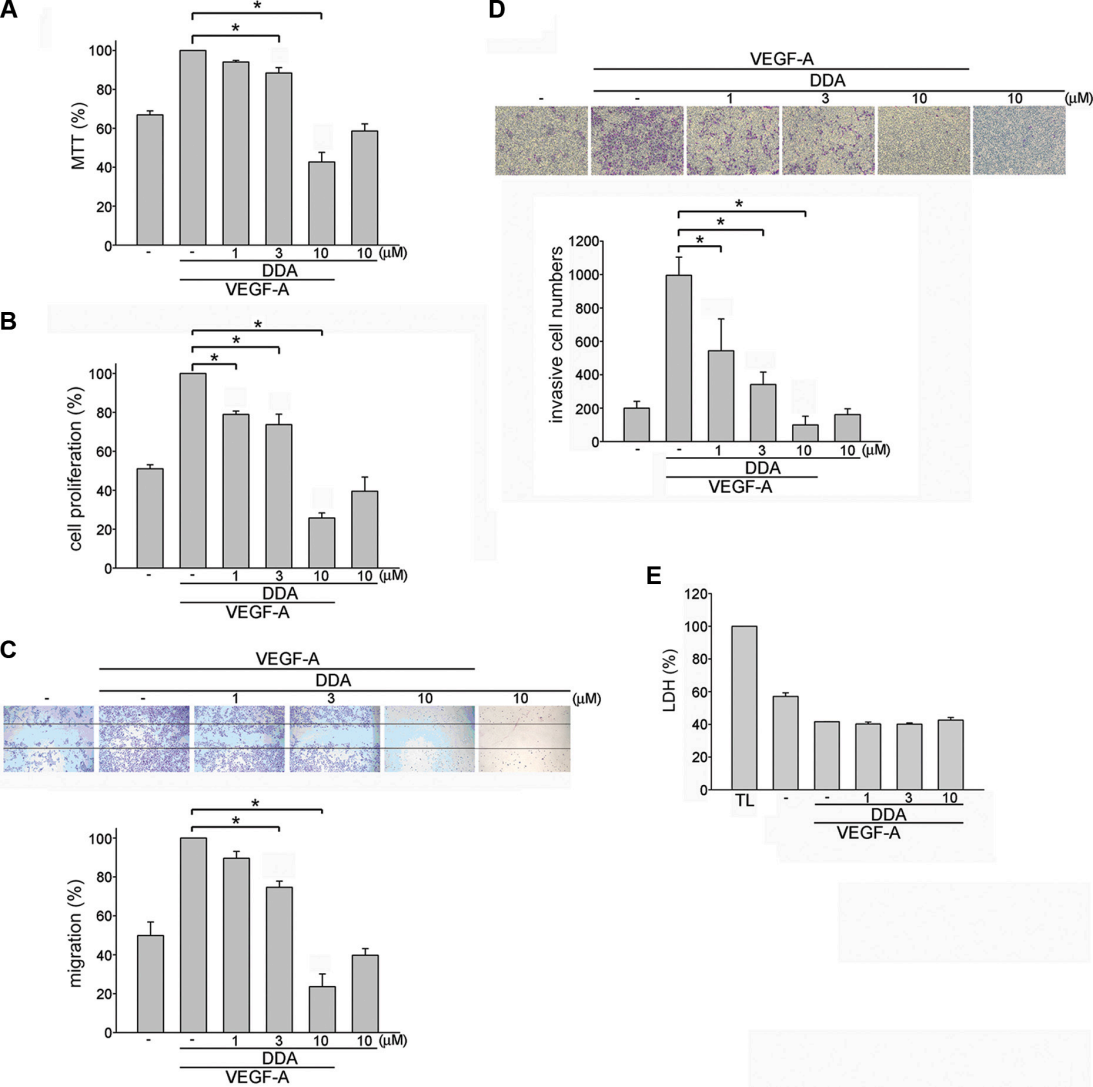
DDA inhibited VEGF-A-induced proliferation, migration and invasion of HUVECs HUVECs were starved in 2% FBS-containing M199 medium without ECGS for 16 h. After starvation, cells were pretreated with indicated concentrations of DDA followed by the stimulation with VEGF-A (25 ng/ml) for another 24 h. Cell viability (**A**) and cell proliferation (**B**) were then determined by MTT assay and BrdU incorporation assay. Each column represents the mean ± S.E.M. of at least three independent experiments performed in triplicate. *p < 0.05, compared with the group treated with VEGF-A alone. (**C**) After starvation, cells were scratched and treated with indicated concentrations of DDA in the presence of VEGF-A for another 24 h. The rate of cell migration was then determined as described in the “*Materials and Methods”* section. Each column represents the mean ± S.E.M. of four independent experiments. *p < 0.05, compared with the group treated with VEGF-A alone. (**D**) After starvation, cells were then seeded in the top chamber in the absence or presence of DDA at indicated concentrations using VEGF-A as chemo-attractant. After 16 h, invaded cells through the gelatin-coated membrane were stained and quantified. Each column represents the mean ± S.E.M. of three independent experiments. *p < 0.05, compared with the group treated with VEGF-A alone. (**E**) After starvation, cells were pretreated with indicated concentrations of DDA followed by the stimulation with VEGF-A (25 ng/ml) for another 24 h. The cytotoxicity of DDA (1, 3, 10 μM) was determined by LDH assay. Cells were also treated with cell lysis buffer (total lysis, TL) to serve as positive control. Each column represents the mean ± S.E.M. of three independent experiments performed in duplicate.

To investigate whether the novel anthraquinone derivative DDA exhibits anti-angiogenic activities, we evaluate DDA's effects on HUVEC proliferation. 2% FBS-containing medium was used to starve HUVECs for 16 h. After starvation, cells were treated with VEGF-A (25 ng/ml) in the absence or presence of the DDA (1–10 μM) for another 24 h. Results from MTT assay demonstrated that DDA reduced cell viability in VEGF-A-stimulated HUVECs (Figure 1A). Results from BrdU labeling analysis showed that DDA reduced cell proliferation in HUVECs after 24 h exposure of VEGF-A (Figure 1B). We next explored whether DDA affects cell motility in HUVECs. Results from wound-healing migration assay showed that HUVEC motility was reduced in the presence of DDA (1–10 μM) using VEGF-A as a chemoattractant (Figure 1C). DDA also reduced cell invasion in VEGF-A-stimulated HUVECs as determined by transwell invasion assay (Figure 1D). In addition, DDA at these concentrations (1–10 μM) barely altered VEGF-A *mRNA* and protein levels in HUVECs (Supplementary Figure 1). We further used LDH assay to determine whether any cytolytic effect contributes to DDA's inhibitory actions in VEGF-A-stimulated HUVECs. LDH release did not significantly increase in HUVECs after 24 h exposure to DDA (1–10 μM) (Figure 1E). Moreover, DDA significantly suppressed bFGF-induced cell proliferation, migration and invasion of HUVECs (Supplementary Figure 2). These results suggest that DDA suppresses angiogenesis through inhibiting cell migration, proliferation and invasion without causing cytolytic effect on HUVECs.

### DDA inhibited HUVEC tube formation and microvessel sprouting in response to VEGF-A

**Figure 2:**
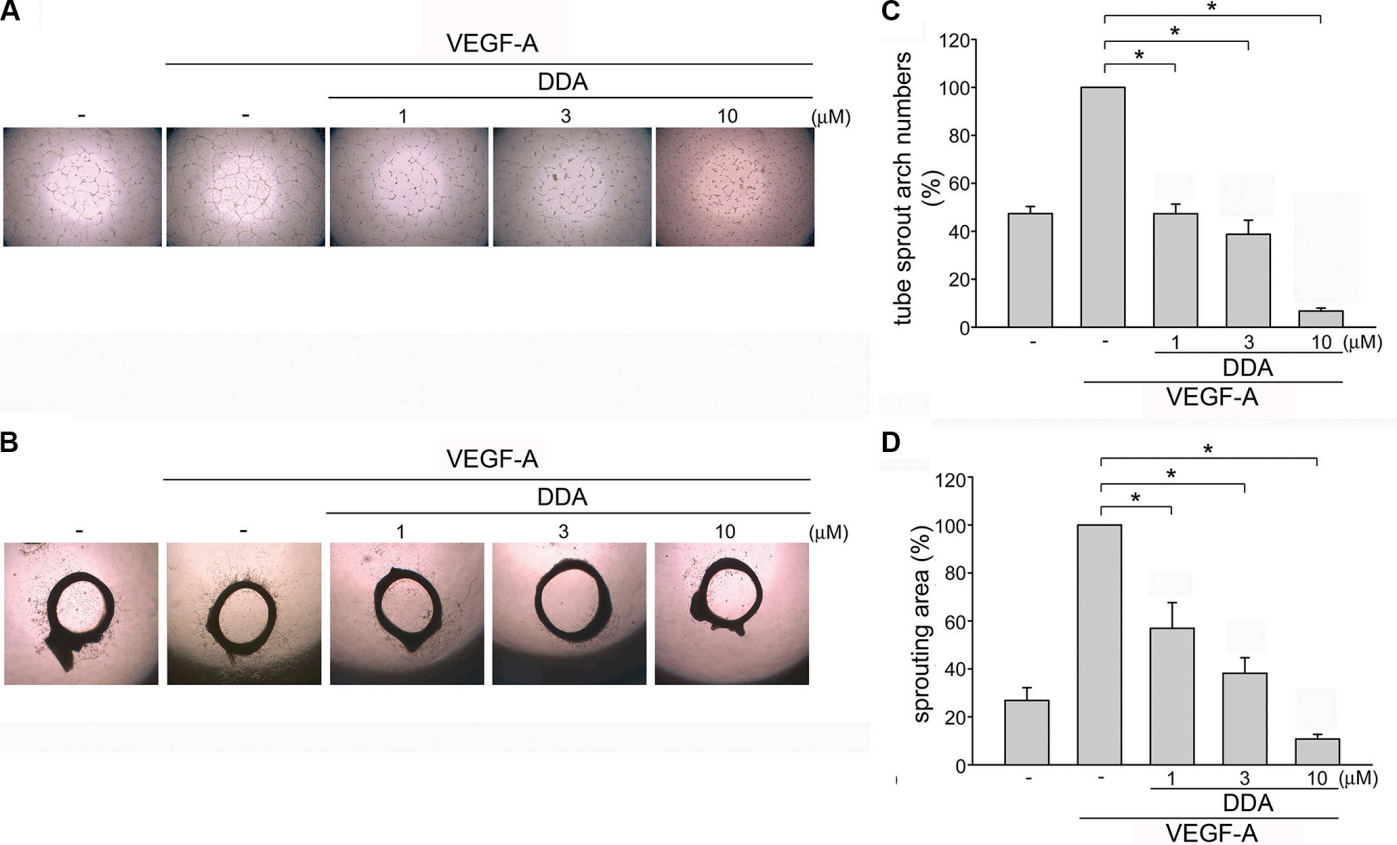
DDA inhibited VEGF-A-induced tube formation of HUVECs and rat aorta ring microvessel sprouting (**A**) HUVECs were seeded on Matrigel in the presence of VEGF-A (25 ng/ml) with or without DDA at indicated concentrations. Cells were then photographed under phase-contrast after 16 h. (B) Rat aortic rings were placed in Matrigel and treated with indicated concentrations of DDA in the presence or absence of VEGF-A (25 ng/ml). The effects of DDA on microvessel sprouting were examined on day 8. Figures shown in (A) and (**B**) are representative of at least seven independent experiments with similar results. (**C**) Bar graphs show compiled data of average sprout arch numbers in (A) (*n* = 8). *p < 0.05, compared with the group treated with VEGF-A alone. (**D**) Bar graphs show compiled data of average microvessels area in (B) (*n* = 6). *p < 0.05, compared with the group treated with VEGF-A alone.

We next assessed DDA's effect on HUVEC tube formation. Results from tube formation assay showed that capillary-like structure was formed by HUVECs after 16 h exposure to VEGF-A (Figure 2A). However, DDA (1–10 μM) reduced VEGF-A-induced capillary-like structure formation (Figure 2C). An *ex vivo* rat aortic ring microvessel sprouting assay was also employed to determine the DDA's anti-angiogenesis effects. As shown in Figure 2B, VEGF-A induced the complex network formation by sprouting microvessels around the aortic rings. However, DDA significantly suppressed this phenomenon (Figure 2D), suggesting that DDA effectively suppresses VEGF-A-induced angiogenesis *ex vivo*.

### DDA reduced VEGF-A- or tumor-induced neovascularization

**Figure 3:**
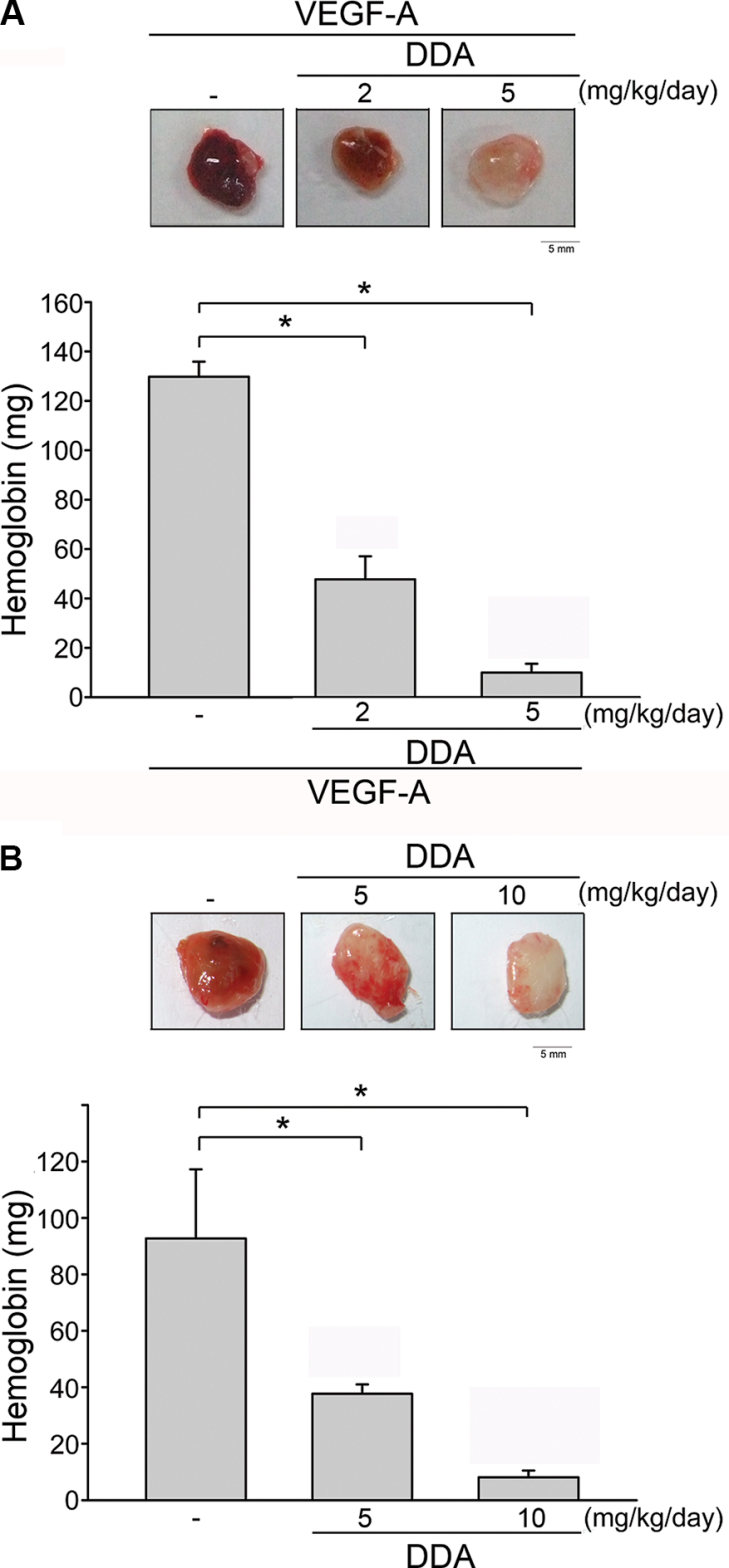
DDA inhibited VEGF-A- or tumor-induced neovascularization (**A**) Matrigel containing VEGF-A and heparin was subcutaneously injected into C57BL/6 mice. Vehicle and DDA (2 or 5 mg/kg/day) was administered intraperitoneally. Hemoglobin levels in the Matrigel plug were quantified 7 days after implantation and shown in the lower panel of the chart. Each column represents the mean ± S.E.M. of six independent experiments. *p < 0.05, compared with vehicle-treated group. (**B**) MDA-MB-231 cells were mixed with Matrigel and injected into both flank sites of male severely combined immunodeficient (SCID) mice. Vehicle and DDA (5 or 10 mg/kg/day) was administered intraperitoneally. Hemoglobin levels in the Matrigel plug were quantified 10 days after implantation. Each column represents the mean ± S.E.M. of six independent experiments. *p < 0.05, compared with vehicle-treated group.

To explore whether DDA exhibits anti-angiogenic effects *in vivo*, the murine matrigel plug model was employed. As shown in Figure 3A, VEGF-A markedly increased microvessel formation in Matrigel plug. In contrast, DDA reduced VEGF-A's enhancing effects on neovascularization over a 7-day period as indicated by the pale color of the plugs (Figure 3A, upper panel). We also determined the hemoglobin content of the plugs to quantify angiogenesis level. As compared with the plugs removed from vehicle-treated control mice, DDA significantly reduced neovascularization in plugs (Figure 3A, lower panel). These observations indicate that the intraperitoneal administration of DDA significantly reduced angiogenesis in this *in vivo* assay. We also used a xenograft tumor-induced angiogenesis model to explore whether DDA inhibits tumor angiogenesis. Matrigel mixed with human breast cancer MDA-MB-231 cells was injected into the flanks of mice. After implantation for 10 days, gel plugs were harvested. As shown in Figure 3B, MDA-MB-231 cells markedly increased neovascularization in the plug while this effect was reduced by DDA (Figure 3B, upper panel). The angiogenesis level was also quantified. As shown in Figure 3B, lower panel, DDA significantly reduced tumor cells-elicited angiogenesis *in vivo*. Moreover, we also evaluated whether DDA affects cell proliferation in MDA-MB-231, PC3 (human prostate cancer cell line), HCT116 (human colorectal cancer cell line), HepG2 (human liver hepatocellular carcinoma cell line) and HS68 (human fibroblast line) cells. Serum-induced cell proliferation of non-tumor HS68 fibroblasts and MDA-MB-231 cells was barely altered after 24 h exposure of 10 μM DDA (Supplementary Figure 4). In contrast, DDA significantly reduced serum-increased HCT116, HepG2 and PC3 cell proliferation (Supplementary Figure 4). These observations suggest that DDA's anti-proliferative activity may vary among different cell types and DDA may target to proliferating endothelial cells to suppress MDA-MB231 tumor cells-induced angiogenesis.

### DDA inhibited VEGFR2 signaling in HUVECs

**Figure 4:**
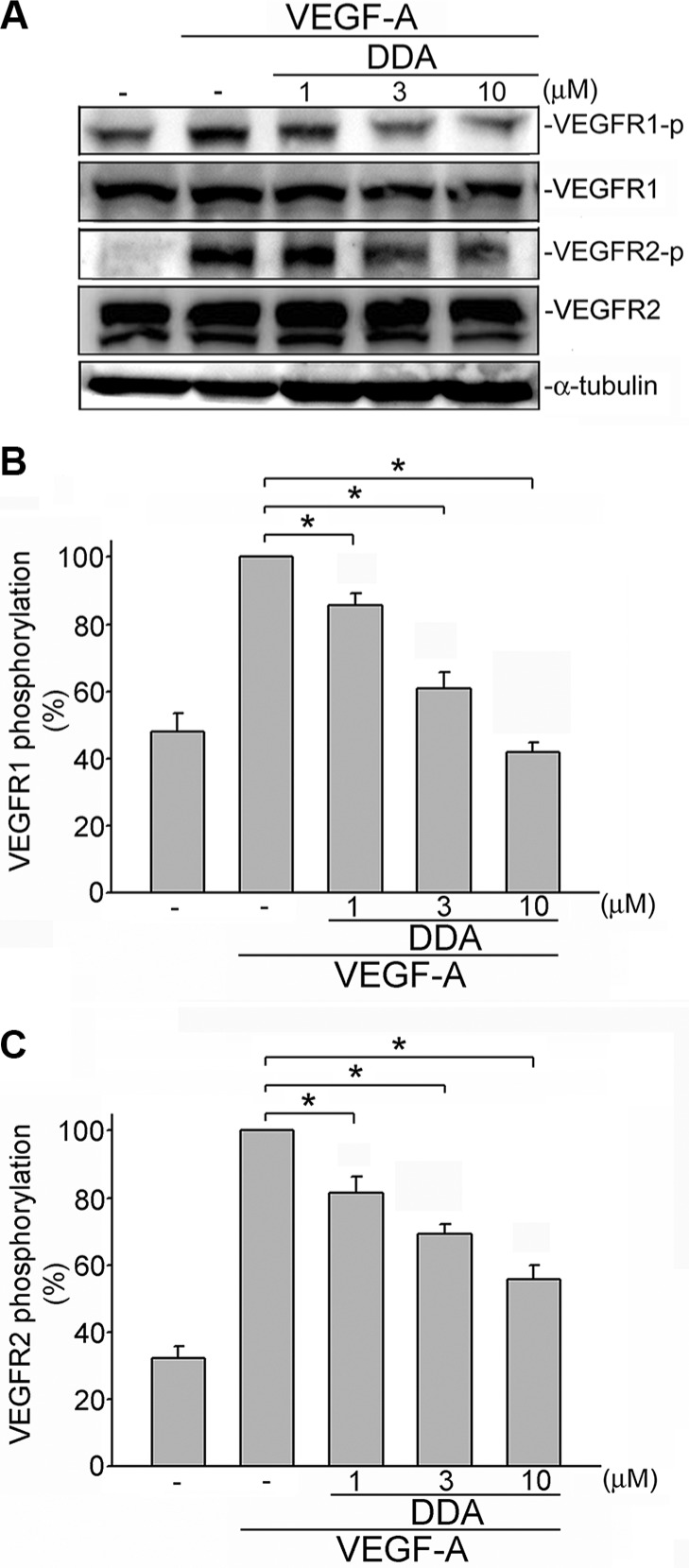
DDA inhibited VEGF-A-induced phosphorylation of VEGFR1 and VEGFR2 in HUVECs (**A**) Cells were pretreated with indicated concentrations of DDA for 30 min, followed by the addition of VEGF-A (25 ng/ml) for another 5 min. Phosphorylation status of VEGFR1 and VEGFR2 were then determined by immunoblotting. The compiled results of VEGFR1 (**B**) and VEGFR2 (**C**) are shown. Each column represents the mean ± S.E.M. of at least five independent experiments. *p< 0.05, compared with the group treated with VEGF-A alone.

**Figure 5:**
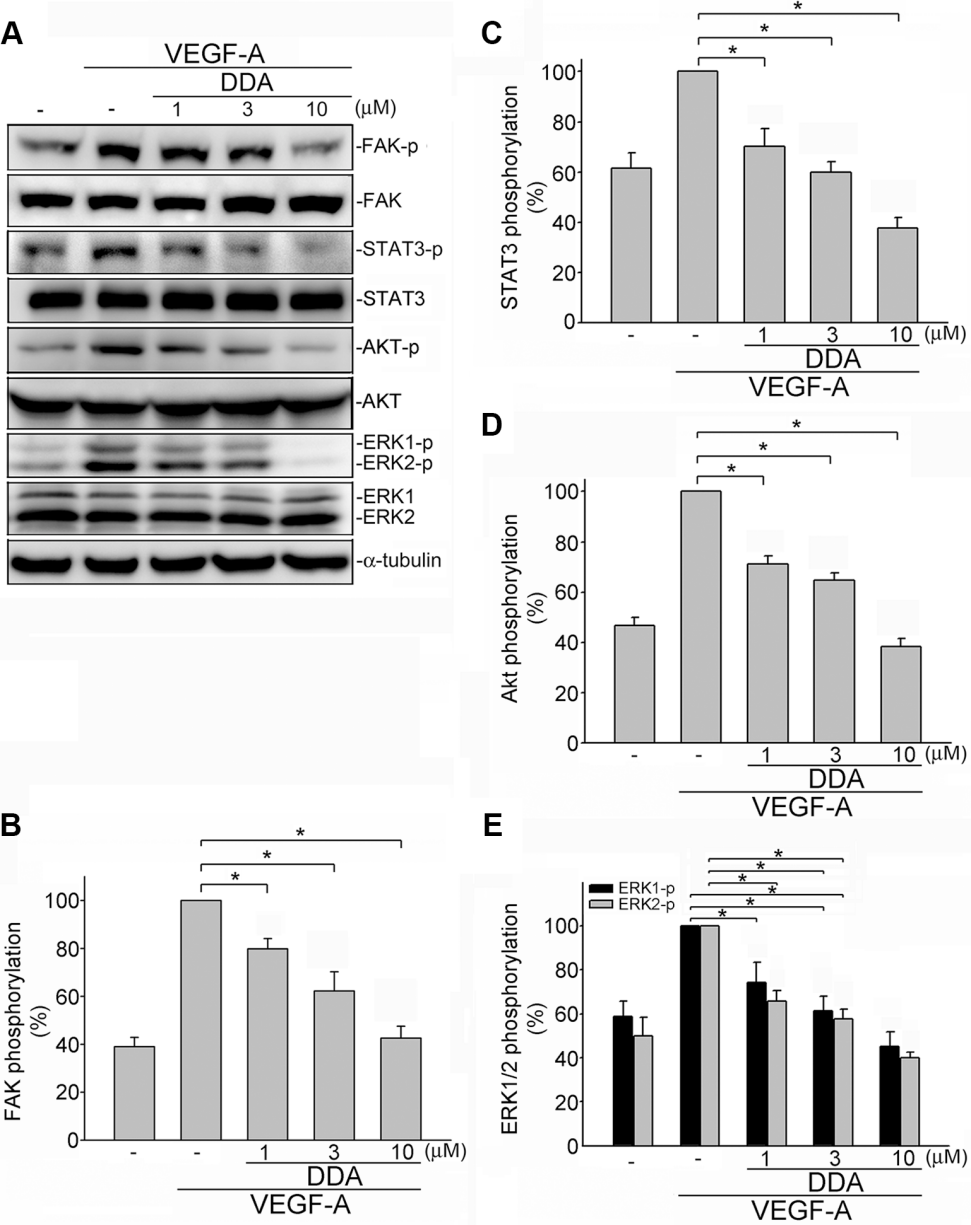
DDA inhibited VEGFR2 signaling pathway in HUVECs (**A**) Cells were pretreated with indicated concentrations of DDA for 30 min, followed by the addition of VEGF-A (25 ng/ml) for another 30 min. Phosphorylation status of FAK, STAT3, Akt and ERK1/2 were then determined by immunoblotting. The compiled results of FAK (**B**), STAT3 (**C**), Akt (**D**) and ERK1/2 (**E**) phosphorylation are shown. Each column represents the mean ± S.E.M. of at least four independent experiments. *p < 0.05, compared with the group treated with VEGF-A alone.

Upon VEGF-A binding, VEGFR2 is phosphorylated, leading to many downstream signaling molecules activation. These signaling molecules such as ERK1/2, FAK, Akt and STAT3 are responsible for endothelial cell proliferation, migration and tube formation. These signaling molecules include ERK1/2, FAK, Akt and STAT3 [11–13, 15, 27]. In addition to VEGFR2, it is reported that VEGFR1 amplifies VEGF-A's responsiveness [28]. We thus determined whether DDA suppresses VEGF-A-induced VEGFR1 and VEGFR2 activation in HUVECs. As shown in Figure 4, DDA inhibited VEGF-A-induced VEGFR1 (Figure 4B) and VEGFR2 (Figure 4C) phosphorylation. Moreover, DDA also suppressed VEGF-A-induced phosphorylation of FAK (Figure 5B), STAT3 (Figure 5C), AKT (Figure 5D) and ERK1/2 (Figure 5E) in HUVECs. Furthermore, DDA also reduced bFGF-induced FGFR phosphorylation in HUVECs beyond its inhibitory effects against VEGF-A-VEGFR2 signaling (Supplementary Figure 3).

### DDA suppressed the formation of NRP-1-VEGFR2 complex in VEGF-A-stimulated HUVECs

**Figure 6:**
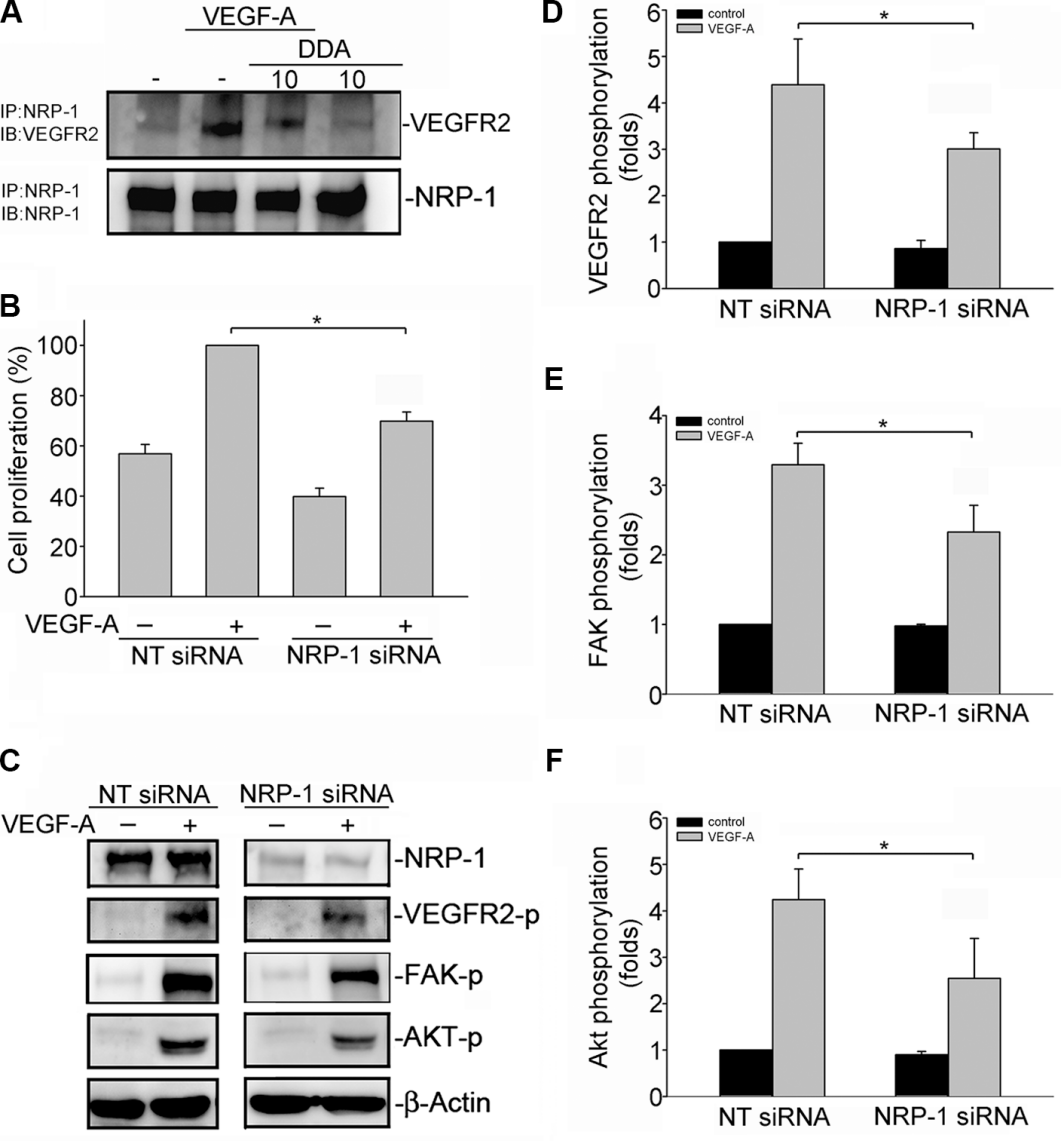
DDA disrupted VEGFR2 and NRP-1 interaction (**A**) Cells were pretreated with indicated concentrations of DDA for 30 min, followed by the addition of VEGF-A (25 ng/ml) for another 10 min. Cells were then harvested and immunoprecipitated with the NRP-1 antibody. The immunoprecipitated complex was then subjected to immunoblotting with an anti-VEGFR2 antibody. Typical bands representative of three independent experiments with similar results are shown. Immunoblotting confirming equal amount of immunoprecipitated VEGFR2 for each sample is shown at the bottom. IP, immunoprecipitation; IB, immunoblotting (**B**) HUVECs were transfected with negative control siRNA or NRP-1 siRNA for 48 h. After transfection, cells were starved for 16 h, and stimulated with VEGF-A (25 ng/ml) for another 24 h. Cell proliferation was determined as described in the “*Materials and Methods”* section. Each column represents the mean ± S.E.M. of four independent experiments. *p < 0.05, compared with the group treated with VEGF-A alone. (**C**) Cells were transfected with negative control siRNA or NRP-1 siRNA for 48 h. After transfection, cells were starved for 16 h, and stimulated with VEGF-A (25 ng/ml) for another 5 (**D**) or 30 (**E**, **F**) min. Phosphorylation status of VEGFR2, FAK and Akt were then determined by immunoblotting. The compiled results of VEGFR2 (D), FAK (E) and Akt (F) are shown. Each column represents the mean ± S.E.M. of four independent experiments. *p < 0.05, compared with the negative control siRNA group treated with VEGF-A alone. NT siRNA, negative siRNA.

Several lines of evidence demonstrated that VEGF-A induced association of neuropilin-1 (NRP1) and VEGFR2 in HUVECs [29]. NRP-1 also contributes to VEGF-A-induced angiogenesis in endothelial cells [29–31]. Co-immunoprecipitation analysis was employed to examine whether DDA affects VEGF-A-induced association of the NRP-1-VEGFR2 complex. As shown in Figure 6A, VEGF-A rapidly induced complex formation of NRP-1 and VEGFR2 in HUVECs. However, DDA (10 μM) caused NRP-1 dissociation from VEGFR2 (Figure 6A). To explore whether NRP-1 contributes to VEGF-A-induced VEGFR2 phosphorylation and subsequent signaling events, HUVECs were transiently transfected with NRP-1 siRNA before VEGF-A treatment. As shown in Figure 6B, NRP-1 siRNA significantly inhibited VEGF-A-induced cell proliferation. NRP-1 siRNA also reduced VEGF-A-induced VEGFR2 (Figure 6D), FAK (Figure 6E) and Akt (Figure 6F) phosphorylation in HUVECs. These observations suggest that DDA's anti-angiogenic actions may also attribute to the disruption of complex formation between NRP-1 and VEGFR2.

### DDA inhibited *in vivo* tumor growth

**Figure 7:**
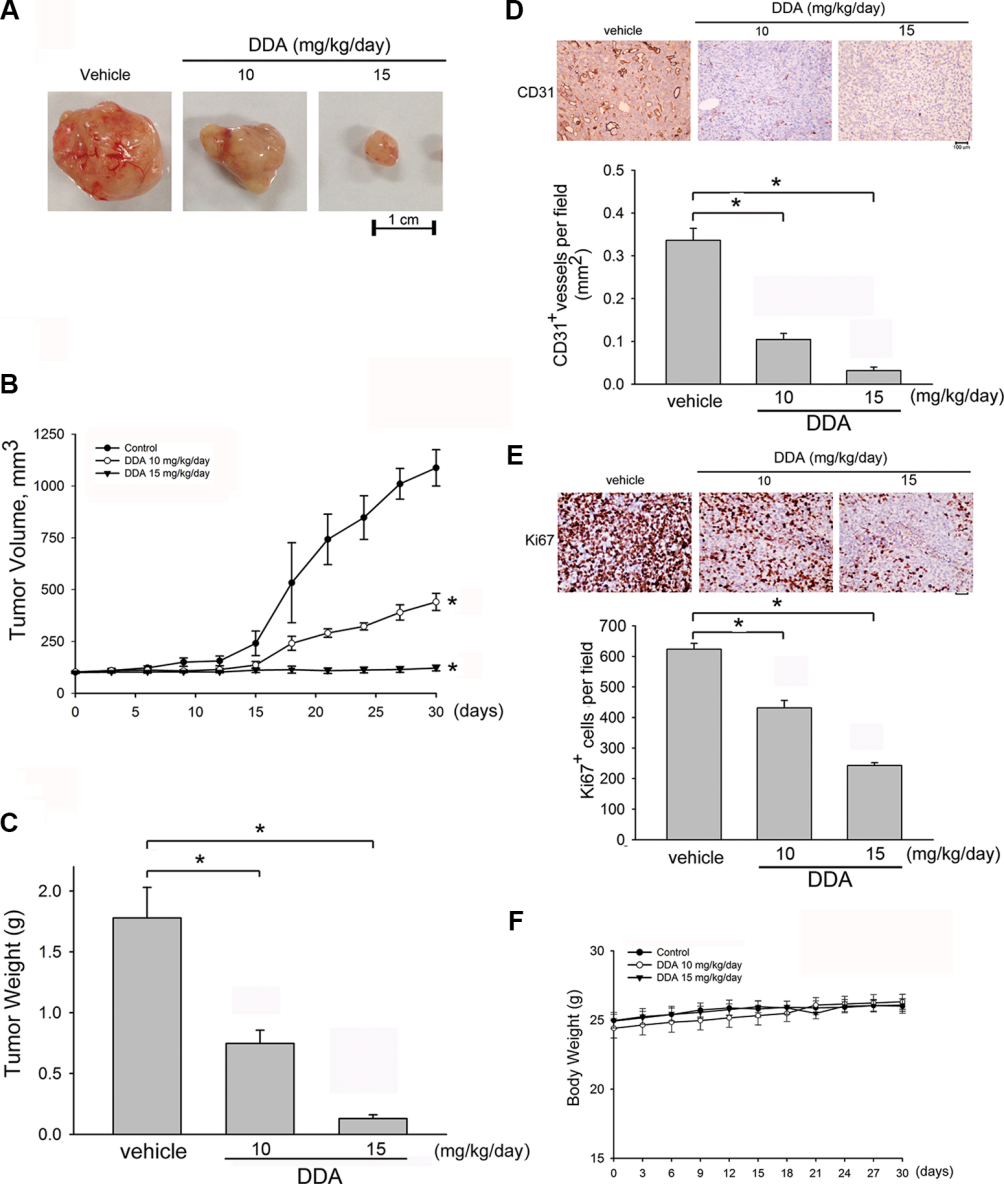
DDA suppressed tumor angiogenesis and tumor growth in a mouse xenograft tumor model (**A**) HCT116 cells were injected into 5-week old nude mice (4 × 10^6^ cells per mice). After solid tumor grew to approximately 100 mm^3^, mice were intraperitoneally administered with vehicle or DDA (10 and 15 mg/kg/day). After treatment for 30 days, solid tumors in DDA-treated mice were significantly smaller than those in control mice (*n* = 5). (**B**) DDA inhibited tumor growth as measured by tumor volume. *p < 0.05, compared with the vehicle-treated group. (**C**) The tumor weight in vehicle- or DDA-treated mice was determined. Bar graphs represent the mean ± SEM of five tumors in each group. *p < 0.05, compared with the vehicle-treated group. The blood vessels (**D**) and proliferative cells (**E**) in solid tumor sections were stained with anti-CD31 (D) and anti-Ki67 (E) antibody. Images of immunohistochemical staining representative of five independent experiments with similar results are shown. Compiled results are shown at the bottom of the chart. Each column represents the mean ± S.E.M. of five independent experiments (*p < 0.05, compared with the vehicle-treated group). (**F**) No significant differences in body weights were found between the control and DDA-treated groups.

A mouse colorectal tumor xenograft model was employed to investigate DDA's inhibitory effects on tumor growth. Intraperitoneal administration of DDA (10 or 15 mg/kg/day) for 30 days markedly reduced tumor volume (Figure 7A). In addition, DDA significantly reduced tumor growth as tumor volume represents tumor growth (Figure 7B) and tumor weight (Figure 7C). We next used an anti-CD31 antibody to stain solid tumor sections to determine whether suppressing angiogenesis contributes to DDA's inhibitory effects on tumor growth. As shown in Figure 7D, the blood vessels in DDA-treated xenografts were less than that in the vehicle-treated xenografts. In addition, the proliferative cells in DDA-treated tumors sections were also reduced as determined by Ki67 staining (Figure 7E). Most cancer therapeutic drugs are reported to have adverse effects or severe cytotoxicity causing body weight loss. Body weight of mice was thus monitored once every 2 days throughout the whole experiment. As shown in Figure 7F, the differences in body weights between vehicle- and DDA-treated groups were not significant. It indicates that DDA suppresses tumor growth through suppressing tumor-induced angiogenesis without causing body weight loss.

## DISCUSSION

Cancer remains a leading cause of mortality worldwide despite incremental advances in surgery, chemotherapy, and radiotherapy. Growing evidence showed that inhibiting VEGF-A-VEGFR2 signaling represents a potential therapeutic strategy in suppressing tumor progression [32–34]. Several angiogenesis inhibitors have already become a clinical anti-tumor strategy in line with chemotherapy and radiotherapy [35]. These observations led to increased efforts to develop novel agents in suppressing angiogenesis. There is increasing evidence demonstrating that anthraquinone derivatives have beneficial effects in the treatment of cancer [36–39]. In the present study, we identified a novel anthraquinone derivative DDA, which exhibits anti-angiogenic properties. We demonstrated that suppressing VEGFR2 signaling contributes to its anti-angiogenic effects. Our results also showed that DDA reduction of tumor growth may be attributed to suppressing angiogenesis in a tumor xenograft model.

Our previous studies demonstrated that PPemd26, a novel anthraquinone derivative, exhibits anti-angiogenic activity through suppression of VEGF-A-VEGFR2 signaling [40]. In this study, DDA, another small molecular anthraquinone derivative with different structure, also inhibited VEGFR2 and its downstream kinases in HUVECs exposed to VEGF-A. These findings suggest that the inhibition of VEGFR2 and subsequent signaling events may contribute to anthraquinone derivatives' anti-angiogenic actions. The underlying mechanisms by which anthraquinone derivatives suppress VEGFR2 signaling remain incompletely understood. There is compelling evidence for an essential role of neuropilin-1 (NRP1) in VEGF-A-induced angiogenesis in endothelial cells [29–31]. It is believed that NRP1 acts as a co-receptor for VEGFR and enhancing VEGF-A signaling [29]. Herzog et al. [41] further demonstrated that NRP1 is essential for VEGF-A-induced FAK phosphorylation, cell migration and angiogenesis of HUVECs. Consistent with these observations, we demonstrated in this study that NRP-1 siRNA reduced VEGFR2, Akt and FAK phosphorylation in VEGF-A-stimulated HUVECs. We also demonstrated that DDA inhibited VEGF-A-induced complex formation between VEGFR2 and NRP-1. The precise mechanisms of DDA in disrupting NRP-1-VEGFR2 complex formation remain to be further investigated. This is the first time that we demonstrated NRP-1 may contribute to an anthraquinone derivative, DDA's actions in suppressing VEGFR2 signaling pathway.

Although VEGF-A-VEGFR2 signaling plays a central role in both physiological and pathological angiogenesis, tumor angiogenesis may also involve other angiogenic growth factors. Recent study demonstrated that other angiogenic factors including bFGF might co-operate with VEGF-A upon angiogenesis [42]. It is plausible that agents targeting not only VEGF-A, but also these angiogenic factors, may be more effective in reducing tumor angiogenesis and metastasis. In addition to VEGF-A, we noted in this study that DDA also exhibited anti-angiogenic properties in HUVECs exposed to bFGF. Further investigations are needed to characterize whether DDA suppresses PDGF- or other angiogenic factor-induced angiogenesis and its underlying mechanisms. Therefore, DDA might act as a multi-target angiogenesis inhibitor.

Although DDA significantly reduced VEGF-A- or MDA-MB-231 cells-induced neovascularization *in vivo*, DDA at 10 μM, however, did not cause cell death in HUVECs and alter MDA-MB-231 cell proliferation. It is likely that DDA targets endothelial cells directly to suppress MDA-MB-231 cells-induced neovascularization. Many studies have highlighted the diverse pharmacological properties of the important pharmacophore, anthraquinone [36–39]. In addition to anti-angiogenic activity [26, 43, 44], suppression of proliferation [45] and induction of apoptosis [46] are also potential mechanisms for anti-cancer effects of anthraquinone derivatives. It raises the possibility that DDA may have additional anti-tumor effects. We demonstrated in the HCT116 colorectal tumor xenograft model that DDA markedly reduced tumor angiogenesis and tumor growth. DDA also significantly inhibited serum-induced proliferation in HCT116, PC3 and HepG2 cells. It is likely that DDA's anti-tumor effects may also attribute to its anti-proliferative activity in tumor cells. However, DDA's anti-proliferative actions on tumor cells might be different among different tumor types. Furthermore, DDA barely affected non-tumor HS68 fibroblast proliferation. The differential effects of DDA' actions in different types of cancer cell remain to be further investigated. Additional works are also needed to characterize the underlying mechanisms by which DDA suppresses tumor cell proliferation.

In conclusion, we showed in the present study that DDA suppresses angiogenesis and tumor growth via, at least in part, inhibiting VEGF-A-VEGFR2 signaling pathways. DDA may serve as a valuable lead compound in the development of anti-cancer therapy.

## MATERIALS AND METHODS

### Reagents

1,5-dihydroxy-4,8-dinitro anthraquinone (DDA), were provided by Dr. Lien JC (Graduate Institute of Pharmaceutical Chemistry, China Medical University, Taichung, Taiwan), and its purity (> 95%) was confirmed by ^1^H-NMR analysis. Antibody against phospho-c-Src Tyr216, phospho-ERK1/2 Tyr204, phospho-Akt Ser473, anti-ERK, anti-FAK, anti-AKT and anti-mouse and anti-rabbit IgG conjugated peroxidase antibodies, rabbit polyclonal antibodies specific for α-tubulin were purchased from Santa Cruz Biotechnology (Santa Cruz, CA). Antibodies against phospho-VEGFR2 Tyr1175, phospho-VEGFR1 Tyr1213, phospho-FAK Tyr397, phosphor-STAT3 Tyr 705, anti-VEGFR2, anti-STAT3 were purchased from Cell Signaling (Danvers, MA). The enhanced chemiluminescence detection kit was from Merck Millipore (Darmstadt, Germany). 3-[4, 5-dimethylthiazol-2-yl]-2, 5-diphenyltetrazolium bromide (MTT), and Toluidine blue O, were from Sigma (St Louis, MO). Medium 199 (M199), RPMI medium 1640, fetal bovine serum (FBS), and all cultured reagents were purchased from Gibco (Grand Island, NY). Endothelial cell growth supplement (ECGS) was from Upstate Biotechnology. Recombinant human VEGF was purchased from R&D Systems (Minneapolis, MN).

### Cell culture

Primary human umbilical vascular endothelial cells (HUVECs), MDA-MB-231 and HCT116 cells were obtained from the Bioresource Collection and Research Center (Hsinchu, Taiwan). The endothelial cells were maintained in M199 medium containing 20% fetal bovine serum (FBS), 5 U/ml heparin, 4 mM L-glutamine, 100 U/ml of penicillin G, 100 μg/ml streptomycin, and 30 μg/ml endothelial cell growth supplements in a humidified 37°C incubator. MDA-MB-231 and HCT116 cell lines were maintained in RPMI1640 containing 10% FCS, 100 U/ml of penicillin G, and 100 μg/ml streptomycin in a humidified 37°C incubator.

### Cell viability assay (MTT assay)

Cell viability was measured by the colorimetric 3-(4,5-dimethylthiazol-2-yl)- 2,5-diphenyl tetrazolium bromide (MTT) assay as described previously [26].

### Cell proliferation assay (BrdU incorporation assay)

HUVECs (2 × 10^4^ per well) were seeded in 96-well tissue culture plates and incubated for 24 h. Cells were then starved in M199 medium containing 2% FBS in the absence of endothelial cell growth supplements for another 16 h. After starvation, cells were pretreated for 30 min with various concentrations of DDA, followed by the stimulation with VEGF-A (25 ng/ml) for another 24 h. Cell proliferation was then determined by bromodeoxyuridine (BrdU) cell proliferation enzyme-linked immunosorbent assay (Millipore, Billerica, MA) based on the colorimetric detection of the incorporation of BrdU, following the manufacturer instructions.

### Wound-healing migration assay

HUVECs were allowed to grow to confluence in six-well tissue culture plates precoated with 0.1% gelatin (Sigma). Cells were then starved with M199 containing 2% FBS for 16 h. After starvation, monolayer HUVECs were wounded by scratching with pipette tips and washed with PBS. M199 containing 2% FBS was added into the wells with or without 25 ng/mL VEGF and various concentrations of DDA. Images of the cells were taken after 24 h treatment. HUVECs were fixed with cold 4% paraformaldehyde and stained with 0.5% toluidine blue in 4% paraformaldehyde. Images were taken by an *OLYMPUS* Biological Microscope digital camera and the rate of cell migration was determined by comparing the sizes of scratch area as a percentage of the values obtained with their respective controls at the beginning of experiments (time 0) using an Image J program.

### Transwell invasion assay

Invasion assay was done as described previously [47]. Briefly, the bottom face of the filter in the transwell plate (Corning, NY, USA) was coated with 0.2% gelatin for 60 min in cell incubator. The bottom chambers were filled with M199 medium containing 2% FBS in the presence of VEGF (25 ng/ml) and the top chambers were seeded with 200 μL M199 medium (without growth factors) and HUVEC (10^5^ cells per well). The top chamber contained vehicle or various concentrations of DDA. After 12 to 16 h of migration, the cells on the top surface of the membrane (non-migrated cells) were scraped with a cotton swab and the cells spreading on the bottom sides of the membrane (invasive cells) were fixed and stained with 0.5% toluidine blue in 4% paraformaldehyde for 30 min. The cells were photographed and quantified by counting the number of stained cells in three random fields at 40× objectives under an *OLYMPUS* Biological Microscope digital camera.

### Matrigel tube formation assay

Matrigel, a basement membrane matrix extracted from Engelbreth-Holm Swarm mouse sarcoma (Becton Dickinson, Mountain View, CA), was polymerized for 30 min at 37°C. HUVECs suspended in M199 containing 2% FBS in the presence or absence of VEGF (25 ng/ml) were seeded onto the Matrigel. They were then treated with DDA at indicated concentrations. After 16 h, cells were photographed using *OLYMPUS* Biological Microscope digital camera at 20× magnification.

### Aortic ring sprouting assay

Assay was performed as previously described [47]. Aortic arch was dissected from 8 to 10-week-old Sprague-Dawley rats. After removing the surrounding fibro-adipose tissues and thoroughly rinsing with M199 culture medium, the aortas were cut into 1 mm ring segments. The aortic rings were immersed in Matrigel in the wells of 48-well plate. VEGF (25 ng/ml) with or without DDA was then added to the wells. The aortic rings were cultured in 37°C with 5% CO_2_ and the cultured medium was changed every 3 days. Growing sprouts of endothelial cells were observed and photographed on day 8. The images were photographed into a computer by using an *OLYMPUS* Biological Microscope, and sprouting area was determined on the computer-digitized images with Image-Pro Plus (Media Cybernetics) software. The analysis of sprouting area was done by an observer who was blinded to the treatment group. All animal study protocol was approved by Laboratory Animal Use Committee of Collage of Medicine, National Taiwan University.

### Suppression of neuropilin-1 (NRP1) expression

For *NRP-1* suppression, predesigned small interfering (si) RNA targeting the human *NRP-1* gene were purchased from Dharmacon. The siRNA oligonucleotides targeting the coding regions of human *NRP-1* messenger (m)RNA were as follows: *NRP-1* siRNA, 5′-GUAUACGGUUGCAAGAUAA-3′; a negative control siRNA comprising a 19 bp scrambled sequence with 3′dT overhangs was also purchased from Dharmacon GE.

### Immunoblotting analysis

Immunoblot analyses were performed as described previously [47]. Briefly, cells were lysed in an extraction buffer containing 15 mM Tris (pH 7.0), 50 mM NaCl, 1 mM PMSF, 5 mM DTT, 1% Triton X-100, 0.05 mM pepstatin A, and 0.2 mM leupeptin. Samples of equal amounts of protein were subjected to SDS-PAGE and transferred onto a polyvinylidene difluoride membrane which was then incubated in a TBST buffer containing 5% BSA. Proteins were visualized by specific primary antibodies and then incubated with HRP-conjugated secondary antibodies. Immunoreactivity was detected using chemiluminescence (ECL) detection system following the manufacturer's instructions. The blot images were quantitated by densitometry using the Image Quant analysis software and normalized with the internal control (α-tubulin).

### Co-immunoprecipitation

HUVECs were grown in 6 cm dishes. After reaching confluence, cells were treated with 25 ng/ml VEGF-A for 5 min. The cells were harvested, lysed in 1 ml of lysis buffer (40 mM Tris-HCl, pH 8.0, 500 mM NaCl, 0.1% Nonidet P-40, 6 mM EGTA, 10 mM β-glycerophosphate, 10 mM NaF, 300 μM sodium orthovanadate, 2 mM phenylmethylsulfonyl fluoride, 10 μg/ml aprotinin, 1 μg/ml leupeptin, and 1 mM dithiothreitol), and centrifuged. The supernatant was immunoprecipitated overnight with specific antibodies against NRP-1 in the presence of protein A/G-agarose beads at 4°C. The immunoprecipitated complex was washed three times with lysis buffer. The samples were fractionated on 8% SDS-PAGE, transferred to a polyvinylidene difluoride and subjected to immunoblotting with antibodies specific for VEGFR2.

### Matrigel plug assay

#### VEGF-induced angiogenesis

Matrigel plug assay was performed as described previously [47]. An aliquot (500 μl) of Matrigel containing VEGF (200 ng/ml) with heparin (20 U) was injected subcutaneously into the dorsal region of 6–8-week-old C57BL/6 mice. Vehicle and DDA (2 or 5 mg/kg/day) was then administered intraperitoneally once a day. After 7 days, the animals were sacrificed, the intact Matrigel plugs were carefully removed, and hemoglobin content in the plugs was determinedwith Drabkin's reagent kit (Sigma-Aldrich) according to the manufacturer's instructions.

### Tumor-induced angiogenesis

MDA-MB-231 breast cancer cells (5 × 10^6^ cells) were mixed with phenol red-free Matrigel and injected into both flanks of severely combined immunodeficient (SCID) mice as described previously [48]. For DDA-treated group, Matrigel was mixed with cells or with medium alone (500 μl) and heparin (20 U). Vehicle and DDA (5 or 10 mg/kg/day) was then administered intraperitoneally once a day. Ten days after implantation, Matrigel plugs were removed and the surrounding tissues trimmed. Hemoglobin levels of the Matrigel plugs were evaluated with Drabkin's reagent kit (Sigma-Aldrich) according to the manufacturer's instructions. The concentration of hemoglobin was calculated based on a set of hemoglobin standards. All animal study protocol was approved by Laboratory Animal Use Committee of Collage of Medicine, National Taiwan University.

### Mouse xenograft model

4-week old BALB/cA nude mice were obtained from National laboratory animal center (National Applied Research Laboratories, Taipei, Taiwan) and maintained on a 12-hour light/dark cycle under controlled temperature (20 + 1°C) and humidity (55 + 5%) in clean specific pathogen free (SPF) rooms. HCT116 cells (4 × 10^6^ cells) in a volume of 0.15 mL PBS were injected subcutaneously into the right flank of each mouse. Once the tumor reached approximately 100 mm^3^, animals were randomized into the control group and the DDA treatment groups. Mice were treated with DDA intraperitoneally once daily for 30 days. Tumors were measured every two-day by a digital caliper. Tumor volume was calculated using the formula *V* (mm3) = [*ab*2] × 0.52, where *a* is the length and *b* is the width of the tumor [49]. At the end of treatment, mice were anesthetized with pentobarbital (1.75 %) with body temperature maintained at 37°C on a thermo-controlled pad. Then the mice were sacrificed and tumors were removed and weighed.

### Immunohistochemistry analysis

Multiple cryosections were obtained from HCT116 tumor xenografts for all immunohistochemical analyses. CD31+ vessel area was assessed using rabbit anti-mouse CD31/PECAM-1 antibody (Abcam) and peroxidase-conjugated goat anti-rabbit IgG (Jackson Research Laboratories) as described previously [50]. Antibody binding was visualized using stable diaminobenzidine. Images were obtained in four different quadrants of each tumor section (2 mm inside the tumor-normal tissue interface) at ×40 magnification. The frozen sections were also used to determine the proliferative cells with an anti-Ki67 antibody (Novus Biologicals, Littleton, CO).

### Statistical analysis

Results are presented as the mean ± S.E. from at least three independent experiments. One-way analysis of variance (ANOVA) was followed by the Newman-Keuls test, when appropriate, to determine the statistical significance of the difference between means. A *p* value of < 0.05 was considered statistically significant.

## SUPPLEMENTARY MATERIALS FIGURES



## Abbreviations

Akt: protein kinase B
bFGF: basic fibroblast growth factor
DDA: 15-dihydroxy-48-dinitro anthraquinone
EGF: epidermal growth factor
ERK: extracellular signal-regulated kinase
FAK: focal adhesion kinase
HUVECs: human umbilical vein endothelial cells
IP: intraperitoneal injection
MTT: 3-[4 5-dimethylthiazol-2-yl]-2 5-diphenyltetrazolium bromide
NRP1: neuropilin-1
PI3K: phosphoinositide 3-kinase
STAT: signal transducers and activators of transcription
VEGF-A: vascular endothelial growth factor-A
VEGFR: vascular endothelial growth factor receptor
