# Supplementary material for: DDA suppresses angiogenesis and tumor growth of colorectal cancer in vivo through decreasing VEGFR2 signaling
Source: Oncotarget. 2016 Aug 9;7(39):63124–37. doi: 10.18632/oncotarget.11152 (PMC5325351; doi:10.18632/oncotarget.11152)
Supplement: Supplementary file 1 [file oncotarget-07-63124-s001.pdf]

DDA suppresses angiogenesis and tumor growth of colorectal cancer *in vivo* through decreasing VEGFR2 signaling

Supplementary Materials

SUPPLEMENTARY METHODS

Reverse-transcription polymerase chain reaction (RT-PCR)

Primers used for amplification of the VEGF-A and GAPDH fragments were as follows: VEGF-A, sense 5′-TCTACCTCCACCATGCCAAG-3′ and antisense 5′-ACCAGGGTCTCGATTGGATG-3′; and GAPDH, sense 5′-AGGGCTGCTTTTAACTCTGGT-3′ and antisense 5′-CCCCACTTGATTTTGGAGGGA-3′. GAPDH was used as the internal control. The PCR was performed with the following conditions: a 5-min denaturation step at 95°C, 30 cycles of a 30-s denaturation step at 95°C, a 30-s annealing step at 57°C, and a 45-s extension step at 72°C to amplify VEGF-A and GAPDH fragments. The amplified fragment sizes for VEGF-A and GAPDH were 130 and 420 bp, respectively. PCR products were run on an agarose gel, stained with ethidium bromide, and visualized by ultraviolet illumination.

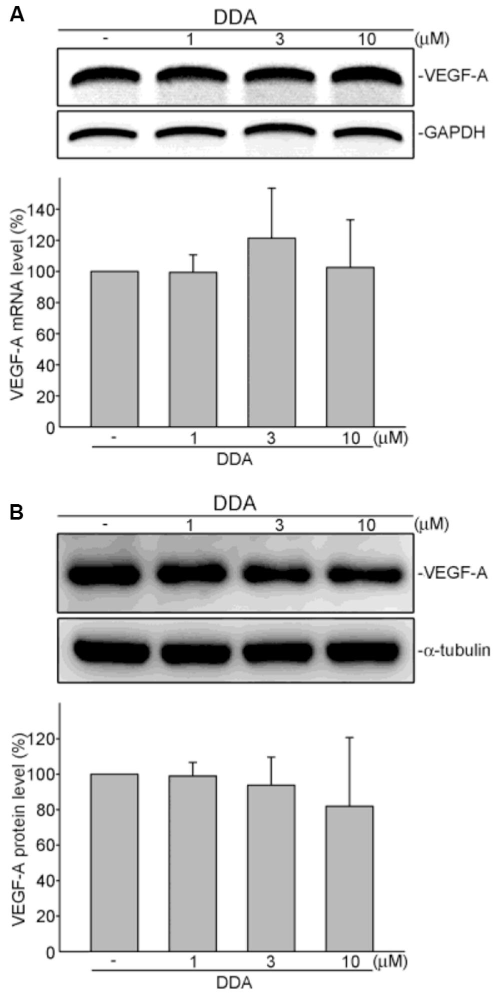

Supplementary Figure S1: Effects of DDA on VEGF-A mRNA and protein levels in HUVECs. Cells were treated with the vehicle or DDA at indicated concentrations for 6 h (**A**) or 24 h (**B**). The VEGF-A mRNA (A) and protein (B) levels were determined by RT-PCR or immunoblotting analysis. Each column represents the mean ± S.E.M. of four independent experiments.

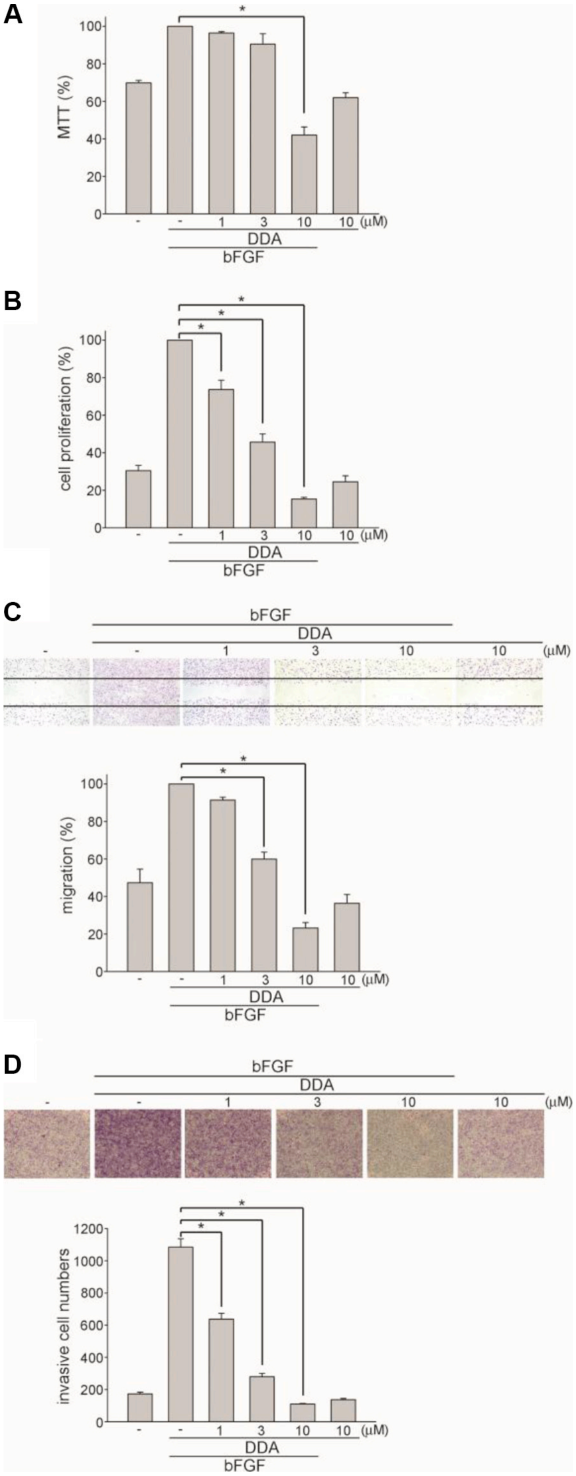

Supplementary Figure S2: DDA suppressed bFGF-induced cell proliferation, migration and invasion of HUVECs. HUVECs were starved in 2 % FBS-containing M199 medium without ECGS for 16 h. After starvation, cells were pretreated with indicated concentrations of DDA followed by the stimulation with bFGF (25 ng/ml) for another 24 h. Cell viability (**A**) and cell proliferation (**B**) were then determined by MTT assay and BrdU incorporation assay. Each column represents the mean ± S.E.M. of at least three independent experiments performed in triplicate. *$p < 0.05$, compared with the group treated with bFGF alone. (**C**) Cells were starved as described in (A). After starvation, cells were scratched and treated with indicated concentrations of DDA in the presence of bFGF for another 24 h. The rate of cell migration was determined. Each column represents the mean ± S.E.M. of four independent experiments. *$p < 0.05$, compared with the group treated with bFGF alone. (**D**) After starvation, cells were then seeded in the top chamber in the absence or presence of DDA at indicated concentrations using bFGF as chemo-attractant. After 16 h, invaded cells through the gelatin-coated membrane were stained and quantified. Each column represents the mean ± S.E.M. of three independent experiments. *$p < 0.05$, compared with the group treated with bFGF.

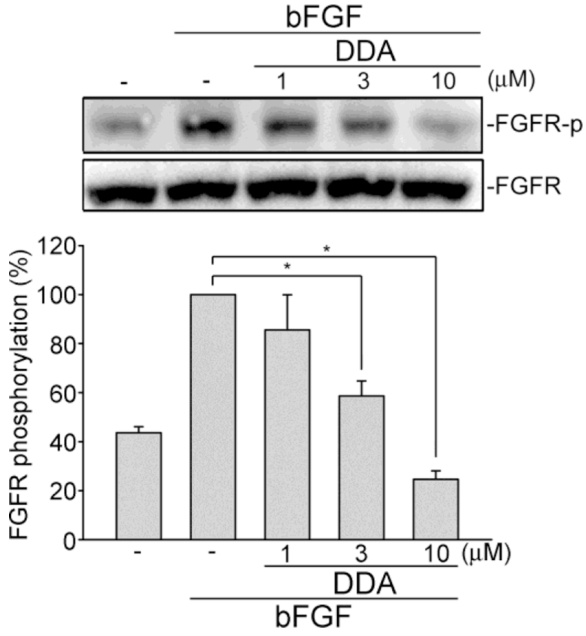

Supplementary Figure S3: DDA suppressed bFGF-induced FGFR phosphorylation in HUVECs. HUVECs were pretreated with indicated concentrations of DDA for 30 min, followed by the addition of bFGF (25 ng/ml) for another 5 min. FGFR phosphorylation status was determined by immunoblotting. Each column represents the mean ± S.E.M. of at least five independent experiments. *$p < 0.05$, compared with the group treated with bFGF alone.

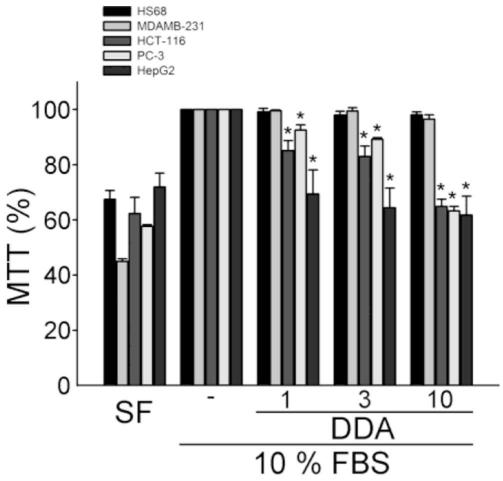

Supplementary Figure S4: Effects of DDA on cell proliferation in HS68, MDAMB-231, HCT116, PC3 and HepG2 cells. After starvation in serum free medium for 24 h, HS68, MDAMB-231, HCT116, PC3 and HepG2 cells were treated with indicated concentrations of DDA in the presence of serum (10% FBS) for another 24 h. Cell viability was then determined by MTT assay. Each column represents the mean ± S.E.M. of at least four independent experiments performed in duplicate. SF: serum free.
